# Effects of a nutritional supplement containing fish protein, vitamin D, and ω3 fatty acids, taken during high-intensity functional training, on physical performance in recreationally trained individuals: a randomized controlled trial

**DOI:** 10.1007/s00394-026-03983-z

**Published:** 2026-05-26

**Authors:** Ioannis Kosmidis, Christina Karpouzi, Anatoli Petridou, Gabriela Voulgaridou, Gregory C. Bogdanis, Vassilis Mougios

**Affiliations:** 1 Laboratory of Evaluation of Human Biological Performance, School of Physical Education and Sport Science at Thessaloniki, Aristotle University of Thessaloniki, Thessaloniki, Greece; 2 Department of Nutritional Sciences and Dietetics, School of Health Sciences, International Hellenic University, Thessaloniki, Greece; 3 School of Physical Education and Sport Science, National and Kapodistrian University of Athens, Athens, Greece

**Keywords:** High-intensity functional training, Multi-ingredient supplement, Proteins, Vitamin D, ω3 fatty acids, Performance

## Abstract

**Purpose:**

Research on supplementation during high-intensity functional training (HIFT) is limited. We examined the effects of a multi-ingredient supplement containing fish protein, vitamin D, and ω3 fatty acids, taken during HIFT, on physical performance in trained individuals.

**Methods:**

Twenty-five recreationally trained participants (14 females, 11 males) underwent 6 weeks of HIFT (3 times/week) while taking each of three supplements, separated by 2-week washout periods in a researcher-blinded, randomized, triple-crossover, and counterbalanced fashion. The supplements and daily doses were (a) 0.6 g fish protein/kg body mass, 21 μg vitamin D, and 1.8 g ω3 fatty acids [including 1.4 g eicosapentaenoic (EPA) and docosahexaenoic acid (DHA)], (b) 0.6 g/kg whey protein, and (c) 0.6 g/kg maltodextrin. Participants followed isoenergetic diets providing 1.0 g protein/kg body mass, 30 μg vitamin D, and 0.2 g EPA and DHA daily. Training variables were assessed during the 1st and 6th weeks of each intervention period. Endurance and strength variables were measured before and after each intervention period. Data were analyzed using 2-way repeated-measure ANOVA (supplement × training).

**Results:**

HIFT augmented workout duration per training session (*p* = 0.007). One-repetition maximum and maximal force of shoulder press, as well as strength endurance of core muscles, increased by 3–6% with training (*p* < 0.001). Supplementation did not affect any of these adaptations.

**Conclusions:**

Six weeks of HIFT improved some training and performance variables in recreationally trained adults. However, increasing the intake of protein, vitamin D, and ω3 fatty acids did not influence the variables assessed.

Trial Registation Number: NCT05402527 (22/4/2022), clinicaltrials.gov.

## Introduction

High-intensity functional training (HIFT) is a training modality based on the CrossFit® principle [1, 2]. This type of high-intensity exercise emphasizes functional movement patterns through both aerobic and resistance exercises, typically performed in short periods, thereby requiring simultaneous engagement of aerobic and anaerobic energy systems. These combined demands promote increases in mitochondrial density, glycolytic capacity, and muscle protein synthesis (MPS) [1–4]. Moreover, HIFT includes multi-joint exercises that are adaptable to any fitness level and elicit greater muscle recruitment than more traditional exercises, enhancing neuromuscular coordination and proprioceptive control [2, 4]. As a result, HIFT has been reported to improve cardiovascular endurance, strength, and flexibility [2, 5–7], while providing a time-efficient alternative to other training methods [3, 5].

Despite extensive research examining the physiological and performance responses to HIFT, only two studies have examined the effects of dietary supplementation during HIFT [8, 9]. The findings show that supplementation during HIFT had no effect [9] or small effects [8]. Interpretation of these findings is constrained by differences in supplement composition (protein versus ω3 and ω6 fatty acids with antioxidant vitamins), study design (cross-over versus parallel-group), and intervention duration (6 versus 8 weeks). These differences preclude meaningful synthesis and highlight the need for further investigation.

Commonly used dietary supplements in sport and exercise include proteins, vitamin D, and ω3 fatty acids [10, 11]. Increased protein intake has been shown to enhance muscle strength [12] and aerobic capacity [13], primarily through its interaction with exercise training to stimulate MPS [12–14]. Athletes and individuals performing resistance training are advised to consume approximately 1.6 g protein/kg body mass/d [11, 12]. Whey protein is the most popular form of protein supplement due to its high nutritional quality [10, 11, 15]. However, fish protein is also an excellent source of high-quality protein with a favorable profile of essential amino acids and a beneficial environmental impact, as it derives from by-products of the fishing industry that are otherwise discarded [16, 17]. Although fish protein shows potential for supporting resistance training adaptations [16], its effects on physical performance remain largely unexplored [17].

Vitamin D exists in two primary forms, D_3_ and D_2_. D_3_ is produced in the skin or obtained through the diet, while D_2_ is obtained only through the diet [18, 19]. Vitamin D plays an important role in the expression of multiple myogenic transcription factors, as well as in myogenesis, MPS, and mitochondrial function. Vitamin D supplementation has been investigated in relation to exercise performance, and Lanteri et al. [18] suggested that a daily dose of 20 μg may represent a minimum threshold for potential performance benefits. However, the findings remain inconsistent [20]. For example, Al-Dujaili et al. [21] reported performance improvements following daily supplementation with 50 μg of vitamin D, whereas Savolainen et al. [22] observed no significant effects in participants with baseline serum 25-hydroxyvitamin D concentrations below 50 nmol/L who received 200 μg of vitamin D daily. These discrepant findings may be partly explained by differences in supplementation dose and duration (2 weeks versus 12 weeks) [20–22]. Moreover, Maughan et al. [23] report that the effects of vitamin D supplementation on muscle function and recovery depend strongly on baseline vitamin D status.

In recent decades, ω3 fatty acids have gained attention as a potential ergogenic aid [24]. These fatty acids are found in fatty fish (e.g., mackerel and salmon), as well as in certain plant oils [24–26]. Evidence suggests that ω3 fatty acids may reduce oxidative stress and improve muscular performance and immune system function [8, 24, 27, 28]. Proposed mechanisms include modulation of MPS, effects on neural tissues, muscle-specific anti-inflammatory actions, and enhancement of mitochondrial function [24, 25]. Intakes of approximately 2 g/d of ω3 fatty acids have been suggested to support training capacity [23]. However, results across studies remain heterogeneous. Whereas performance enhancements have been reported under certain supplementation protocols [8, 24, 27, 28], other studies employing a wide range of ω3 doses [0.2–7.0 g/d of eicosapentaenoic (EPA) and docosahexaenoic acid (DHA)] and intervention durations (1 week to 3 years) have reported insufficient evidence for performance benefits [25, 26]. Importantly, baseline ω3 status may affect responsiveness [25].

Multi-ingredient supplements are popular among athletic populations [29]. The literature reports a variety of ingredients in such supplements, the most common being creatine, amino acids, caffeine, vitamin D, and polyunsaturated fatty acids [11, 29]. Combinations of these ingredients may have additive or synergistic effects on exercise performance. However, empirical findings are inconsistent. In their meta-analysis, Naclerio and Larumbe-Zabala [30] concluded that whey protein showed stronger evidence of beneficial effects on maximal strength during resistance training when consumed as part of a multi-ingredient supplement containing creatine than when consumed alone. In contrast, O’Bryan et al. [11] found that multi-ingredient supplementation during resistance training was not superior to protein-only supplementation in increasing strength. These divergent findings may be explained by differences in supplement composition across studies, as Naclerio and Larumbe-Zabala [30] primarily examined whey protein with creatine or other protein sources, whereas O’Bryan et al. [11] included supplements combining protein with other ingredients such as vitamin D, fatty acids, and carbohydrates. Despite these differences, both studies indicate that multi-ingredient supplements have a positive effect on strength.

Considering the limited research on protein supplementation in HIFT, as well as on fish protein, vitamin D, and ω3 fatty acid supplementation during training in general, the present study aimed to compare the effects of a multi-ingredient nutritional supplement (containing fish protein, vitamin D, and ω3 fatty acids), whey protein (as a positive comparator), and maltodextrin (as a negative comparator) during HIFT on physical performance in recreationally trained individuals. We hypothesized that, compared with maltodextrin, the multi-ingredient supplement and whey protein would improve training outcomes related to physical performance.

## Materials and Methods

### Study design and ethics

**Fig. 1:**
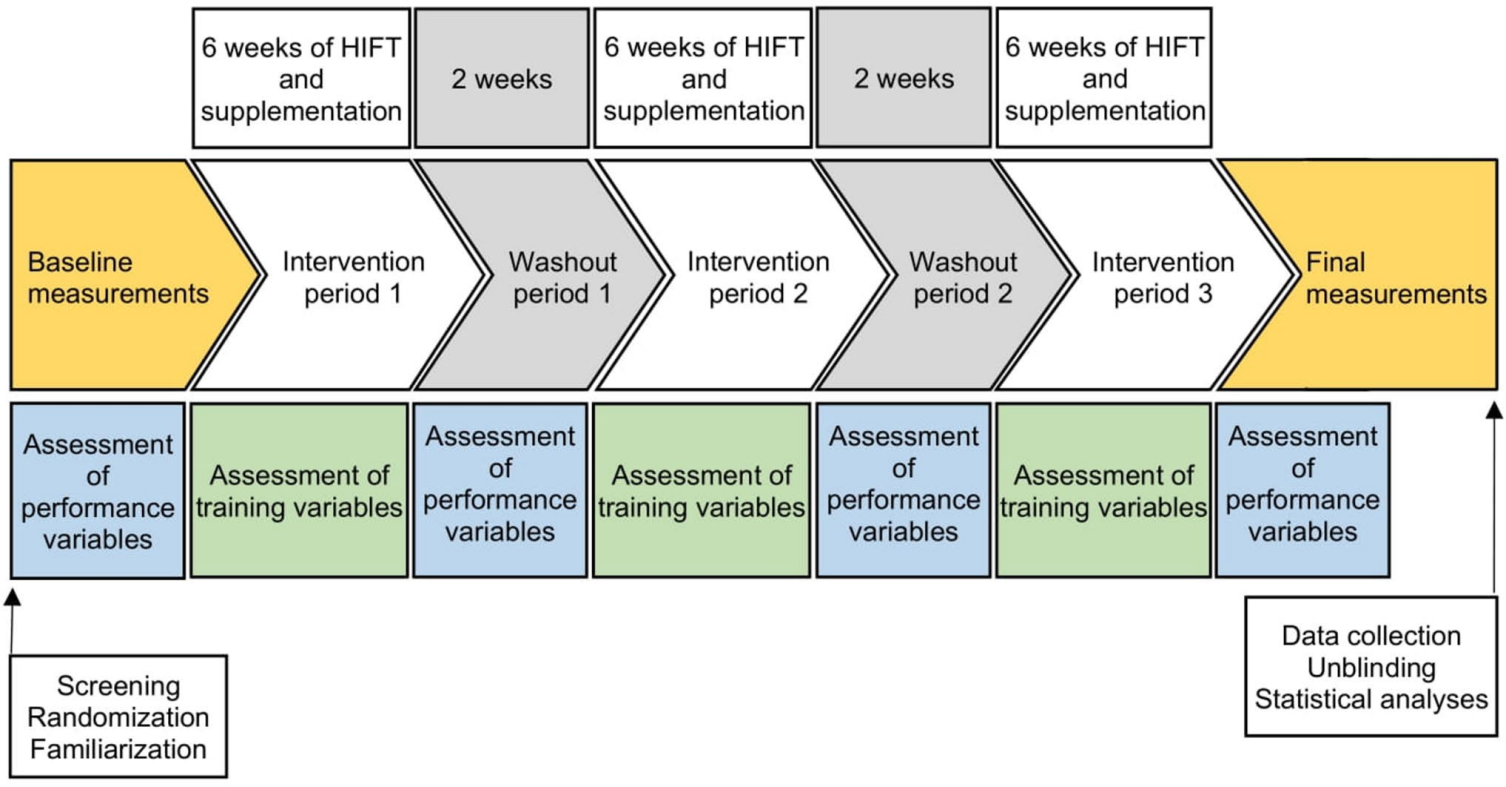
Study design

A randomized, triple-crossover, and counterbalanced study was performed to evaluate the effects of dietary supplementation on physical performance in recreationally trained individuals. The experimental protocol is presented in Fig. 1. The effects of each of the three supplements were evaluated during 6 weeks of HIFT (3 intervention periods in total) with 3 sessions per week and a washout period of 2 weeks between intervention periods (2 washout periods in total). The duration and frequency of HIFT were based on the existing literature, which recommends a minimum of 4 weeks with at least 2 sessions per week for significant effects on physical fitness and sport-specific performance [1]. The duration of washout was based on two pilot studies conducted prior to this study. In each pilot study, two female and two male volunteers consumed either the multi-ingredient supplement (in one study) or the whey protein supplement (in the other study), as described below. Plasma amino acid and whole-blood ω3 fatty acid concentrations were measured as described [31, 32] before supplementation, after 6 weeks of supplementation, and after a 2-week washout period. Τhe results showed an increase in the total plasma amino acid concentration and (in the case of the multi-ingredient supplement) total blood ω3 fatty acid concentration with supplementation, followed by a return to baseline after washout, supporting the adequacy of a 2-week washout period.

Thus, the study lasted 22 weeks (excluding the days spent on the initial and final assessments), from December 6, 2021, to May 8, 2022. Measurements of the training variables were conducted during the 1st and 6th weeks of each intervention period, whereas performance measurements were made before the 1st intervention period, during both washout periods, and after the 3rd intervention period.

The study was blinded on the part of the outcome assessors, investigators, HIFT instructors, and supplement distributors. Participants were not blinded due to differences in the taste, smell, and form of the supplements. (The multi-ingredient supplement was a combination of strawberry-rhubarb-flavored powder and capsules, whey protein was a strawberry-flavored powder, and maltodextrin was a tasteless powder.) Participants were instructed not to disclose which supplement they received during each intervention period to the outcome assessors, investigators, and HIFT instructors.

The study was approved by the Ethics Committee of the School of Physical Education and Sport Science at Thessaloniki, Aristotle University of Thessaloniki (approval number EC-31/20-10-2020), was registered in the ClinicalTrials.gov database (NCT05402527/22/4/2022), and was carried out in compliance with the Declaration of Helsinki.

### Participants

The sample size was determined a priori using the G*Power software (version 3.1.9.2; Kiel University, Kiel, Germany; RRID: SCR_013726). To identify significant effects with a medium effect size of 0.059 (as η^2^ for factorial ANOVA) [33], α of 0.05, power of 0.8, and a correlation coefficient between repeated measures of 0.5, a minimum of 19 participants was needed. However, in view of potential dropouts (particularly due to the COVID-19 pandemic, which was still widespread during the study), we opted to recruit 25 participants.

The recruitment of volunteers was performed by the first author among gym clients, through social networks, from personal acquaintances, and by posting a call on the website of the School of Physical Education and Sport Science at Thessaloniki. Volunteers completed a questionnaire, provided by email, with questions regarding the inclusion and exclusion criteria. The inclusion criteria were age 18–35; regular training (mixed endurance and resistance training 3–6 times a week, at least 50 min per session) for the past 4 months; clearance from a pathologist or cardiologist to perform maximal exercise; and mixed isoenergetic diet over the past month. The exclusion criteria were smoking; musculoskeletal injury; chronic disease; fish, oyster, or milk allergy; pregnancy; lactation; regular use of prescription medication or supplements that might affect muscle function or recovery over the past month; and religious or intermittent fasting. Based on this screening procedure, 14 female and 11 male participants were recruited. Their age was 24.0 ± 4.5 years, body mass 68.1 ± 13.4 kg, height 1.71 ± 0.11 m, and body mass index 23.0 ± 2.1 kg/m^2^ (all mean ± SD).

The participants were invited to a first visit to the laboratory, during which they were given detailed written and oral instructions concerning the study procedures, dates, measurement venues, benefits, and potential risks. Then, they provided written informed consent, and their body weight and height were measured. Body weight was assessed using an electronic scale (Seca, Hamburg, Germany) to the nearest 0.1 kg with minimal clothing. Height was measured to the nearest 0.5 cm using a stadiometer fitted to the balance. Finally, participants were familiarized with the performance measurements of the study.

Following the first visit, one of the authors generated the allocation sequence by drawing lots. The same person dispensed the supplements to the participants throughout the study.

### Composition of the multi-ingredient supplement

The multi-ingredient supplement consisted of two parts: (a) a powder containing protein, vitamin D, and ω3 fatty acids, and (b) soft gelatin capsules containing additional ω3 fatty acids. The powder was prepared from cod filleting side streams (fins and tails that contained meat and some bones) and hydrolysed cod collagen (skin and some bones). To this, vitamin D_3_ (cholecalciferol) and strawberry-rhubarb powder (for flavor) were added. The capsules contained 1 g of herring oil.

### Supplementation

During each day of the three 6-week intervention periods, participants received (a) the multi-ingredient supplement (Seagarden, Avaldsnes, Norway) in quantities providing 0.6 g protein/kg body mass, 21 μg vitamin D, 0.5 g ω3 fatty acids (EPA and DHA) from the powder, and 1.3 g ω3 fatty acids from 4 capsules (containing 0.9 g EPA and DHA), (b) a whey protein supplement (Warriorlab Complete Whey, Warriorlab, Athens, Greece) in quantities providing 0.6 g protein/kg, or (c) a maltodextrin supplement (Warriorlab Maltodextrin, Warriorlab) in quantities providing 0.6 g carbohydrate/kg. The amino acid profiles of supplements (a) and (b), according to the manufacturers, are provided in Table 1. The supplements were stable for at least 12 months when stored at up to 25 °C.

**Table 1:** Percent amino acid composition of the multi-ingredient and whey protein supplements

Amino acid	Multi-ingredient supplement	Whey protein
Alanine	7.2	5.2
Arginine	7.2	2.6
Aspartate + Asparagine	1.6	9.9
Cysteine	0.3	2.3
Glutamate + Glutamine	14.3	17.2
Glycine	10.5	2.1
Histidine	1.8	1.8
4-Hydroxyproline	9.5	0.0
Isoleucine	4.2	5.6
Leucine	7.1	10.7
Lysine	7.6	9.1
Methionine	2.9	1.9
Phenylalanine	3.6	3.5
Proline	5.8	5.9
Serine	4.7	4.6
Threonine	4.0	6.8
Tryptophan	0.0	1.9
Tyrosine	3.0	3.1
Valine	4.7	5.8
Essential amino acids*	35.9	47.1
Branched-chain amino acids**	16.0	22.1

^*^Histidine, isoleucine, leucine, lysine, methionine, phenylalanine, threonine, tryptophan, and valine. **Isoleucine, leucine, and valine

The supplements were stored in a dry, cool place, away from direct sunlight, and were consumed in two equal daily doses, spaced approximately 12 h apart, one in the morning (09:00–12:00) and one in the evening (21:00–24:00). The timing of supplementation relative to the training sessions was not controlled, as previous research has shown it does not significantly affect long-term adaptations to resistance training [34], and no comparable data are available for HIFT. The powders were dissolved in tenfold quantities of fruit juice containing 11% carbohydrate. Participants were asked to record their doses in diaries prepared for them. No supplements were taken during the washout periods.

### Dietary control

Participants received mixed, isoenergetic dietary plans throughout the study, starting one week before and ending one week after supplementation. The dietary plans were modified every 6 weeks for the sake of variety. They were designed to provide, daily, 1.0 g protein/kg (reaching 1.6 g/kg when consuming the multi-ingredient supplement or whey protein), about 55% of energy from carbohydrate (taking into account the quantity of fruit juice used to dissolve the supplements), and about 35% of energy from fat, including 0.2 g from EPA and DHA (reaching 1.6 g when consuming the multi-ingredient supplement). The dietary plans also provided 30 μg of vitamin D (reaching 51 μg when consuming the multi-ingredient supplement). Nutrient values were based on the USDA FoodData Central database [35] and nutrition information labels on packaged foods. Each week, participants reported their estimated compliance with the dietary plan as a percentage.

### Training protocol

Training was carried out in three well-equipped commercial gyms, each providing the same group programs. Participants were instructed to participate in any of the available HIFT sessions three times per week, with the flexibility to alternate between four HIFT programs throughout the study. This flexibility was intended to enhance the ecological validity of the study by mirroring the typical behavior of individuals who engage in exercise programs. All programs included multimodal patterns of movement, combining both endurance and strengthening exercises with equipment such as TRX, BOSU, kettlebells, and barbells. Workouts lasted approximately 45 min, including a 5-min warm-up and a 5-min cool-down, and could be modified according to the progression of the participants’ performance.

All sessions were supervised by certified instructors who were thoroughly briefed by the investigators in advance. Instructors also checked the proper application of the exercise protocol, including the “work to momentary failure” principle. Before the beginning of the 1st intervention period, participants were instructed to avoid any intense training [that is, above 76% of maximal heart rate (HRmax)] [36] during the remaining four days of the week. Furthermore, we asked them to record their sessions in diaries provided for this purpose. During the washout periods, the participants continued their usual exercise routines.

### Training variables during HIFT sessions

Training variables were assessed at the gyms during a session of the 1st and 6th weeks of each intervention period. Heart rate (HR) was monitored using the Polar Team Pro GPS telemetric system (Polar, Kempele, Finland). The software provided the following metrics: workout duration, average HR, workout time in each of 5 HR zones based on percentages of HRmax (zone 1, 50.1–60; zone 2, 60.1–70; zone 3, 70.1–80; zone 4, 80.1–90; zone 5, 90.1–100), total energy expenditure, and training load score (an index of the strenuousness of the training session, calculated based on its intensity and duration) [37].

### Performance variables

Performance variables were measured at the Laboratory of Evaluation of Human Biological Performance at the beginning of the study (within the two weeks preceding it), during the washout periods, and within one week after the end of the last intervention period. Performance measurements were conducted over two days to ensure that participants could perform the tests to their full potential and were scheduled at least three days after the last training session of each intervention period to avoid acute effects on the outcome measures. On day one, we assessed, in sequence, the force–velocity relationship of knee extensors and flexors, maximal dynamic strength of shoulder press, and aerobic capacity. On day two, we evaluated strength endurance of knee extensors and flexors, force–velocity relationship of shoulder press, and strength endurance of core muscles in that order. At the beginning of each assessment day, participants completed a general warm-up consisting of 5 min of moderate-intensity cycling, followed by dynamic stretching targeting the muscle groups scheduled for assessment.

#### Maximal dynamic strength of shoulder press

Maximal dynamic strength of the shoulder press was assessed using the one-repetition maximum (1RM) test, following the guidelines of the US National Strength and Conditioning Association [38]. Participants received standardized instructions on proper shoulder press technique using a Smith machine, with the bench’s back inclined to a near-vertical position to support the head and torso. Feet were placed flat on the floor, with knees bent at approximately 90°. After a warm-up with light weights, participants performed a set of 10 repetitions at 50% of their estimated 1RM, followed by 4–6 repetitions at 75%, and then 2–4 repetitions at 85%. The load was then progressively increased by 2.5–10 kg until only one repetition could be completed. A rest period of 4 min was maintained between sets and single efforts. Participants were verbally encouraged to perform at their best, and each effort was supervised by the investigator to ensure safety and correct execution.

#### Force–velocity relationship of shoulder press

The force–velocity relationship of the shoulder press was evaluated using a Smith machine equipped with a linear position transducer (Tendo Power Analyzer System v. 314, Trencin, Slovak Republic) to measure peak force and peak velocity during the shoulder press. The participants were positioned as described above. Female participants lifted loads approximating 45, 55, 65, and 75%, in that order, of their 1RM, while male participants lifted loads approximating 30, 45, 60, and 75%, in that order, of their 1RM. At each load, participants performed three consecutive explosive repetitions, with 3-min rests between sets. The average peak force and velocity of the three repetitions for each load were used to generate a force–velocity graph. From this, the y-intercept (representing maximal force at zero velocity) and the x-intercept (representing maximal velocity at zero force) were determined.

#### Strength endurance of core muscles

Strength endurance of core muscles was evaluated by counting the number of consecutive sit-ups performed within 60 s. Participants began in a supine position with arms crossed over the chest, knees bent, and feet held in place by an investigator, who provided verbal encouragement and counted the repetitions. A repetition was considered valid when the participants raised the torso and touched the knees with the elbows [6].

#### Force–velocity relationship of knee extensors and flexors

The force–velocity relationship of the knee extensors (quadriceps) and flexors (hamstrings) was evaluated using an isokinetic dynamometer (HUMAC NORM 770, Stoughton, MA) by measuring maximal concentric contractions at angular velocities of 60, 120, 180, and 240°/s. Participants were seated upright with the torso supported by the vertical backrest and secured with chest and thigh straps. The dynamometer’s axis was adjusted so that the center of motion of the lever arm was aligned with the flexion–extension axis of the knee joint. The range of motion was kept between 0 and 90°. Participants completed three consecutive maximal concentric extensions and flexions at each velocity, with 1 min of rest from one angular velocity to the next. Strong verbal encouragement was provided throughout. Peak torque was defined as the highest value recorded for knee extension and flexion at each angular velocity.

#### Strength endurance of knee extensors and flexors

Strength endurance of the knee extensors and flexors was evaluated using a fatigue protocol on the same isokinetic dynamometer as above. The protocol involved 30 consecutive maximal concentric extensions and flexions at an angular velocity of 180°/s, during which the participants received strong verbal encouragement. Positioning of the participant and range of motion were as described above. Strength endurance was calculated as the ratio of total work performed during the last 15 repetitions to that of the first 15 repetitions, multiplied by 100 (fatigue index).

#### Aerobic capacity

Aerobic capacity was evaluated by measuring maximal oxygen uptake (VO₂max) using a graded maximal exercise test on a horizontal treadmill, connected with an ergospirometer (Jaeger Oxycon Pro, Würzburg, Germany). For female participants, the test began at 5 km/h, with the speed increasing by 1 km/h every minute until exhaustion. Male participants followed the same protocol, with the exception that they started at 6 km/h. Oxygen uptake was recorded breath-by-breath and averaged over every five breaths. HR was continuously monitored using a Polar HR monitor (Polar, Kempele, Finland). The criteria to declare VO₂max were any two of the following: a plateau in oxygen uptake despite an increase in speed (< 150 mL/min or < 2.1 mL/kg/min), a respiratory exchange ratio exceeding 1.10, and an HR within 10 beats/min of the age-predicted maximal (HRmax = 220 – age).

### Adverse events

Adverse events were monitored using an active reporting approach. During the study, participants were systematically asked, on a weekly basis, whether they had experienced any adverse events. In some cases, these events prevented participants from completing certain measurements. Analysis of these adverse events revealed that nine participants fell ill due to COVID-19, two contracted bronchitis, one developed sinusitis, one developed tonsillitis, one developed fever of unknown etiology, and two sustained injuries (one during a HIFT session and the other during an outdoor activity). It is unlikely that these adverse events were due to supplementation. Additionally, it is unlikely that they affected the validity of the study, as they were evenly distributed between supplements.

### Handling of missing data

Among the 25 participants, 11 had complete datasets, 8 missed between one and three measurements due to adverse events or other personal circumstances, and 6 withdrew between weeks 6 and 16. We aimed to perform an intention-to-treat (ITT) analysis, which utilizes data from all enrolled participants to reduce bias that may arise from analyzing only data from the participants who adhered to and completed the treatment as originally designed [per-protocol (PP) analysis] [39, 40]. However, ITT analysis faces challenges in handling missing data resulting from dropouts, no-shows, or technical issues. In ITT analysis, missing outcomes are estimated using available data through various methods [41]. In the present study, four participants dropped out between weeks 6 and 9, resulting in their data being fewer than or equal to the missing data. Because of the complex design of the study (administering 3 different supplements during different periods), we deemed it too arbitrary to supplement the missing data of these participants by any method. As a result, we excluded these four participants from the analysis. For two participants who withdrew from the study in weeks 15 and 16 (completing two-thirds of the study) and for eight participants with one to three missing data, we used a combination of imputation methods. Specifically, we replaced missing data at the beginning of the study with the value from the next measurement, missing data in the middle of the study with the mean of the previous and next values, and missing data at the end of the study with the value of the previous measurement. The causes of the missing data were unrelated to the research questions. Thus, these data can be considered as “missing completely at random,” [41] and including the inferred data in the analysis is expected to produce unbiased conclusions.

**Fig. 2:**
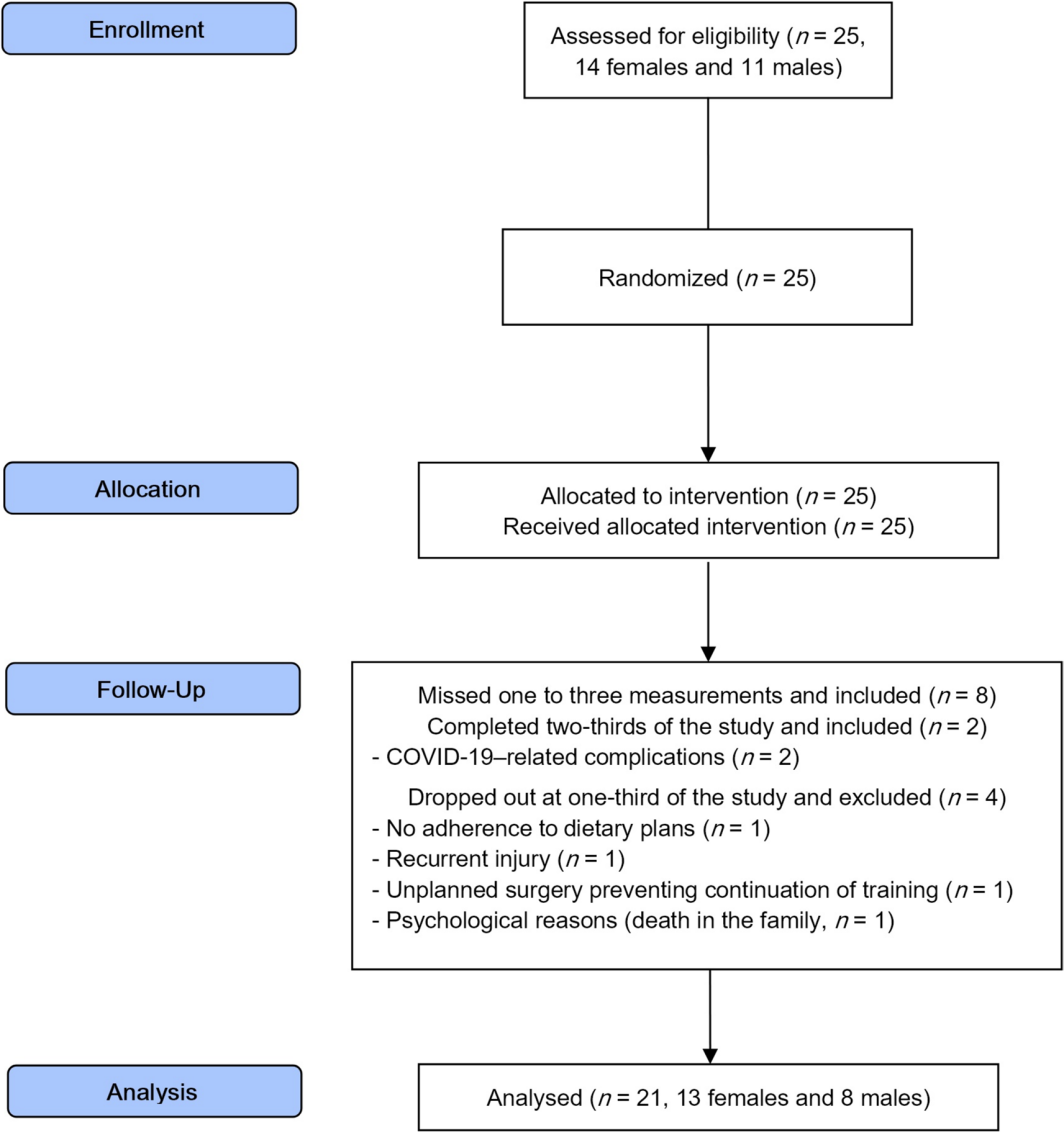
CONSORT 2010 flow diagram

In summary, we adopted a mixed model of data handling, offering a balance between the ITT and PP approaches. As a result, 21 participants (13 female and 8 male) were included in the analysis (Fig. 2).

### Statistical analysis

Descriptive data are presented as the mean ± SD. The distribution of supplements between intervention periods for the 21 participants that were finally included in the analysis was compared by the χ^2^ test. Repeated-measure analysis of variance (ANOVA) was performed to examine differences in the number of training sessions, doses of supplement taken, and adherence to the dietary plans during the intake of each supplement. To analyze the effects of supplementation and training (that is, time) on the outcome measures of the study, we initially performed a 3-way ANOVA with three levels of supplementation (multi-ingredient supplement, whey protein, maltodextrin), two levels of training (pre and post), two levels of sex (females and males), and repeated measures on supplement and training. The results did not produce any interaction of sex with the other two factors; only well-documented differences between sexes, (e.g., higher 1RM in males than females) were observed. No different responses of the two sexes to the interventions were identified. Therefore, we performed 2-way ANOVA with three levels of supplementation (multi-ingredient supplement, whey protein, maltodextrin), two levels of training (pre and post), and repeated measures on both factors as our main analysis. A significant main effect of supplementation indicates differences between supplements, based on the combined pre- and post-training values for each supplement. A significant main effect of training reflects an impact of HIFT regardless of supplementation. A supplement × training interaction indicates differential effects of supplements on the adaptations to training and thus represents the most valid criterion of the effectiveness of a supplement. Sphericity was tested with Mauchly’s test and, when violated, the Greenhouse–Geisser correction was applied. Effect sizes (ES) for main effects and interaction were determined as partial η^2^ and were classified as small (0.01–0.058), medium (0.059–0.137), or large (> 0.137) according to Cohen [33]. Correlations between variables were examined with Pearson’s correlation analysis. Statistical significance was set at α = 0.05. All statistical analyses were performed using SPSS (version 27.0.1; IBM SPSS Statistics, Armonk, NY, USA; RRID:SCR_002865), and all figures were generated using OriginPro (version 2025b, 10.2.5.212; OriginLab Corporation, Northampton, MA, USA; RRID:SCR_014212).

## Results

### Effect of confounding variables

We assessed four possible confounding variables: randomization of supplementation, number of training sessions during the intake of each supplement, doses of each supplement taken, and adherence to the dietary plans during the intake of each supplement. None of these variables showed statistically significant differences between supplements. Adherence rates were 89 ± 11% for training sessions, 94 ± 7% for supplement intake, and 75 ± 11% for dietary plans.

### Training variables

**Fig. 3:**
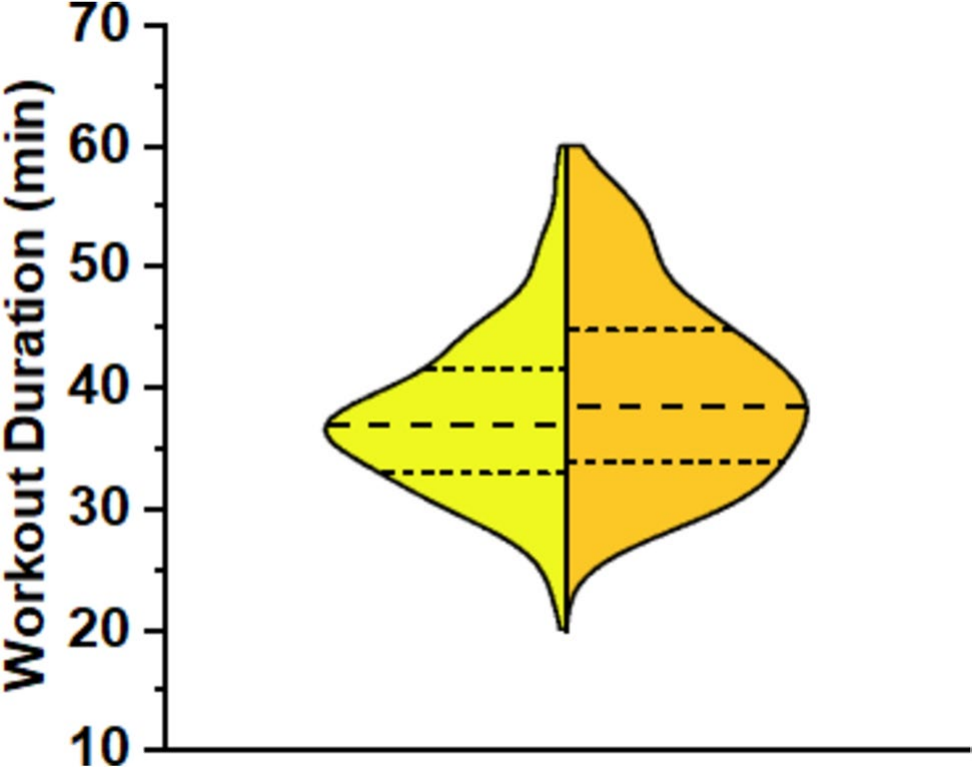
Violin plot of workout duration (*p = 0.007) in a training session during the 1st (yellow) and 6th (orange) week of HIFT, independent of supplementation. The width of the violin represents the frequency distribution of the data. The central dashed line represents the median, while the upper and lower dashed lines delimit the interquartile range

Table 2 presents the descriptive and inferential statistics of the training variables during a HIFT session in the 1st week and a HIFT session in the 6th week of each intervention period. There was a significant main effect of training on workout duration, which increased by 7% (from 37.5 ± 6.4 to 39.9 ± 7.6 min, *F*_1, 20_ = 9.142, *p* = 0.007*,* 95% CI 45.8 to 249.5, Fig. 3), with a large ES (partial η^2^ = 0.314). No main effects of supplement or supplement × training interaction were observed.

**Table 2:** Descriptive (mean ± SD) and inferential statistics of training variables during a HIFT session of the 1st and a HIFT session of the 6th (final) week of each intervention period

Variable	Multi-ingredient supplement		Whey protein		Maltodextrin		Main effect of supplement			Main effect of training			Supplement x training interaction		
	1st week	6th week	1st week	6th week	1st week	6th week	*F*	^*p*^	ES	*F*	^*p*^	ES	*F*	^*p*^	ES
Workout duration (min)	37.1 ± 6.7	39.9 ± 7.7	38.9 ± 7.4	39.0 ± 7.9	36.4 ± 5.0	40.9 ± 7.3	0.044	0.957	0.002	9.142	**0.007**	0.314	1.658	0.203	0.077
Average HR (% HRmax)	76 ± 5	76 ± 6	76 ± 5	76 ± 5	78 ± 5	77 ± 4	2.126	0.133	0.096	2.045	0.168	0.093	0.215	0.807	0.011
Time in HR zone 1 (%)	6.3 ± 6.7	6.5 ± 10.0	5.7 ± 5.7	6.2 ± 5.4	3.6 ± 5.5	5.4 ± 5.9	1.140	0.330	0.054	0.813	0.378	0.039	0.215	0.808	0.011
Time in HR zone 2 (%)	20.4 ± 12.8	21.0 ± 13.0	20.5 ± 9.9	22.9 ± 13.1	17.3 ± 11.5	18.5 ± 10.1	1.695	0.197	0.078	0.743	0.399	0.036	0.156	0.856	0.008
Time in HR zone 3 (%)	31.0 ± 9.0	30.4 ± 12.1	34.0 ± 13.1	31.4 ± 10.3	30.3 ± 11.6	32.7 ± 7.2	0.419	0.660	0.021	0.015	0.903	0.001	0.982	0.383	0.047
Time in HR zone 4 (%)	32.1 ± 15.1	32.5 ± 16.7	30.0 ± 13.3	29.4 ± 16.4	36.4 ± 15.5	32.3 ± 12.7	1.222	0.336	0.053	0.333	0.570	0.016	0.667	0.519	0.032
Time in HR zone 5 (%)	9.4 ± 10.5	8.3 ± 12.4	8.9 ± 11.1	9.1 ± 12.1	11.9 ± 13.8	10.0 ± 12.4	0.583	0.563	0.028	0.616	0.442	0.030	0.311	0.734	0.015
Energy expenditure (kcal)	364 ± 101	391 ± 125	380 ± 109	373 ± 103	373 ± 90	405 ± 121	0.957	0.753	0.014	1.242	0.055	0.172	0.429	0.335	0.053
Training load score	70 ± 19	73 ± 26	73 ± 20	72 ± 23	75 ± 20	79 ± 16	0.285	0.393	0.046	4.144	0.278	0.058	1.124	0.654	0.021

ES, effect size (as partial η^2^); HR, heart rate. Boldface indicates significant (*p* ≤ 0.05)

### Performance variables

**Fig. 4:**
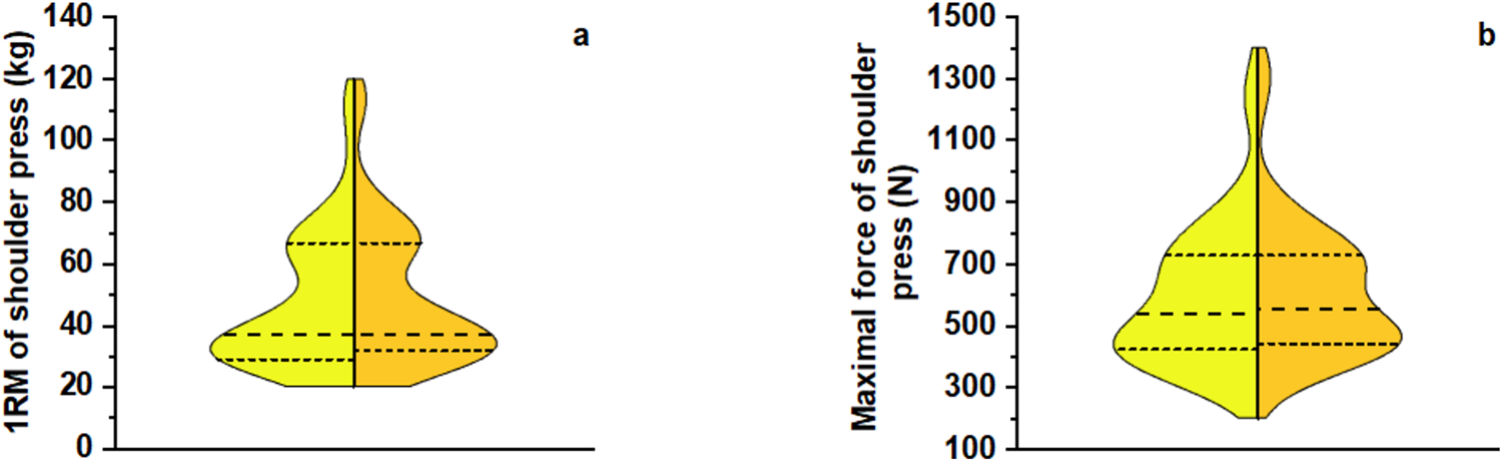
Violin plots of **(a)** 1RM and **(b)** maximal force of the shoulder press before (yellow) and after (orange) each intervention period, independent of supplementation. The width of each violin represents the frequency distribution of the data. The central dashed line represents the median, while the upper and lower dashed lines delimit the interquartile range. *p < 0.001

Table 3 presents the descriptive and inferential statistics of the performance variables before and after each intervention period. A significant increase was observed in both 1RM and maximal force of the shoulder press with training. Specifically, 1RM increased by 4% (from 46.8 ± 21.4 to 48.8 ± 22.2 kg, *F*_1, 20_ = 36.445, *p* < 0.001, 95% CI 1.3 to 2.6, Fig. 4a), and maximal force increased by 3% (from 592 ± 227 to 611 ± 219 N, *F*_1, 20_ = 15.223, *p* < 0.001, 95% CI 9.2 to 30.3, Fig. 4b), both with large ES (0.646 and 0.432, respectively).

**Table 3:** Descriptive (mean ± SD) and inferential statistics of performance variables before and after each intervention period

Variable	Multi-ingredient supplement		Whey protein		Maltodextrin		Main effect of supplement			Main effect of training			Supplement × training interaction		
	Pre-training	Post-training	Pre-training	Post-training	Pre-training	Post-training	F	^p^	ES	F	^p^	ES	F	^p^	ES
1RM of shoulder press (kg)	47.1 ± 22.1	48.7 ± 22.7	46.2 ± 20.6	49.0 ± 22.7	47.2 ± 22.6	48.7 ± 22.2	0.172	0.842	0.009	36.445	** < 0.001**	0.646	0.872	0.426	0.042
Maximal force of shoulder press (N)	582 ± 231	611 ± 229	587 ± 222	596 ± 218	605 ± 240	626 ± 219	2.271	0.116	0.102	15.233	** < 0.001**	0.432	0.272	0.763	0.013
Maximal velocity of shoulder press (m/s)	2.89 ± 0.89	2.75 ± 0.85	2.85 ± 0.88	2.98 ± 0.93	2.78 ± 0.80	2.63 ± 0.96	4.389	**0.019**	0.180	1.317	0.256	0.062	0.767	0.471	0.037
Strength endurance of core muscles (sit-ups/min)	40 ± 10	43 ± 9	41 ± 8	42 ± 9	40 ± 9	44 ± 10	0.352	0.705	0.017	37.897	** < 0.001**	0.655	0.712	0.497	0.034
Peak torque of knee extensors 60°/s (Nm)	162 ± 49	160 ± 48	159 ± 48	158 ± 48	164 ± 52	158 ± 48	0.451	0.640	0.022	4.434	**0.048**	0.181	0.324	0.725	0.016
Peak torque of knee flexors 60°/s (Nm)	98 ± 29	98 ± 28	101 ± 28	100 ± 30	99 ± 29	97 ± 28	1.834	0.173	0.084	1.668	0.211	0.077	0.316	0.731	0.016
Peak torque of knee extensors 120°/s (Nm)	137 ± 39	134 ± 41	131 ± 39	134 ± 39	137 ± 42	135 ± 40	1.428	0.252	0.067	0.100	0.755	0.005	0.924	0.405	0.044
Peak torque of knee flexors 120°/s (Nm)	86 ± 28	87 ± 28	89 ± 26	89 ± 27	88 ± 28	86 ± 25	2.793	0.073	0.123	0.437	0.516	0.021	0.412	0.665	0.020
Peak torque of knee extensors 180°/s (Nm)	119 ± 36	120 ± 39	115 ± 34	119 ± 35	120 ± 39	115 ± 35	1.192	0.314	0.056	0.935	0.345	0.045	0.692	0.507	0.033
Peak torque of knee flexors 180°/s (Nm)	76 ± 25	76 ± 26	79 ± 26	78 ± 25	77 ± 26	74 ± 23	3.851	**0.030**	0.161	1.898	0.184	0.087	0.737	0.485	0.036
Peak torque of knee extensors 240°/s (Nm)	104 ± 32	105 ± 37	100 ± 30	103 ± 32	103 ± 36	103 ± 31	1.488	0.238	0.069	4.201	0.054	0.174	0.719	0.493	0.035
Peak torque of knee flexors 240°/s (Nm)	66 ± 22	64 ± 23	66 ± 21	68 ± 23	66 ± 23	64 ± 21	2.817	0.072	0.123	0.310	0.584	0.015	1.334	0.273	0.063
Fatigue ratio of knee extensors (%)	69 ± 8	69 ± 8	68 ± 7	68 ± 9	68 ± 8	67 ± 7	3.148	0.054	0.136	0.239	0.630	0.012	0.269	0.765	0.013
Fatigue ratio of knee flexors (%)	67 ± 8	64 ± 10	65 ± 10	64 ± 9	65 ± 9	66 ± 10	0.302	0.741	0.015	1.902	0.183	0.087	0.503	0.609	0.025
VO_2_max (mL/kg/min)	45.3 ± 7.4	45.7 ± 7.5	45.5 ± 8.2	45.6 ± 7.8	45.7 ± 7.4	45.5 ± 7.7	0.004	0.996	0.000	0.132	0.720	0.007	0.135	0.874	0.007

ES, effect size (as partial η^2^); 1RM, one-repetition maximum. Boldface indicates significant *(p* ≤ 0.05)

**Fig. 5:**
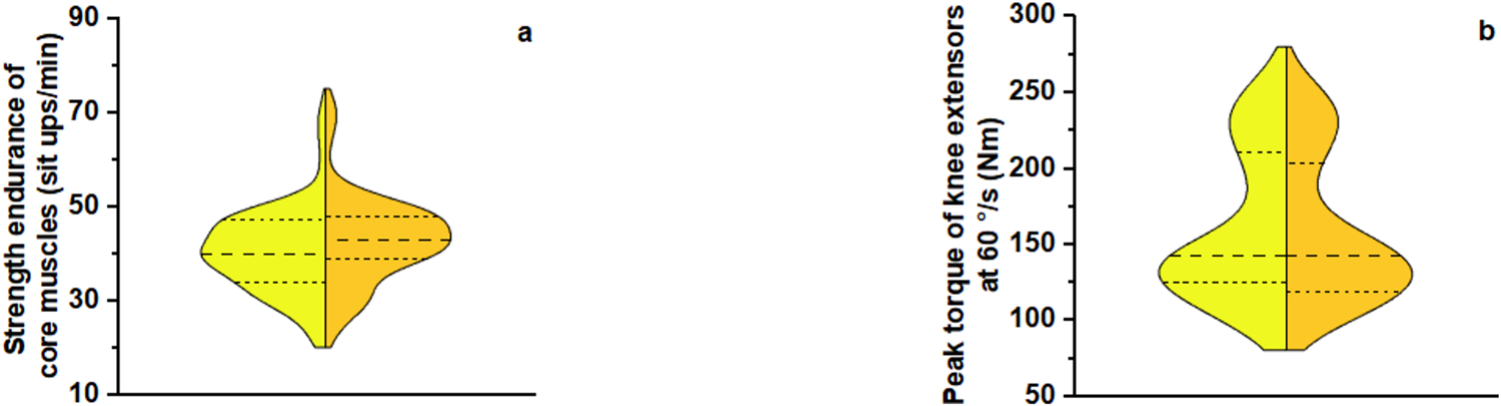
Violin plots of **(a)** strength endurance of core muscles (*p < 0.001) and **(b)** peak torque of the knee extensors at 60°/s (*p = 0.048) before (yellow) and after (orange) each intervention period, independent of supplementation. The width of each violin represents the frequency distribution of the data. The central dashed line represents the median, while the upper and lower dashed lines delimit the interquartile range

Finally, the strength endurance of core muscles increased with training by 6% (from 40 ± 9 to 43 ± 9 sit-ups/min, *F*_1, 20_ = 37.897, *p* < 0.001, 95% CI 1.6 to 3.3, Fig. 5a), with a large ES (0.655), whereas the peak torque of the knee extensors at 60°/s decreased by 2% (from 161 ± 49 to 159 ± 47 Nm, *F*_1, 20_ = 4,434, *p* = 0.048, 95% CI –5.1 to –0.02, Fig. 5b), with a large ES (0.181). No main effects of supplement or supplement × training interaction were observed.

## Discussion

In this triple-crossover study, we compared the effects of a multi-ingredient nutritional supplement (containing fish protein, vitamin D, and ω3 fatty acids) with those of whey protein (as a positive comparator) and maltodextrin (as a negative comparator) during 6 weeks of HIFT on physical performance in recreationally trained individuals. The use of relatively understudied supplementation sources in the multi-ingredient supplement, combined with a relatively novel training approach that emphasizes multi-joint exercises mimicking real-life movements, strengthens the originality and impact of our study. Our findings show that workout duration per training session, 1RM and maximal force of shoulder press, and strength endurance of core muscles increased with exercise training. In contrast, peak torque of the knee extensors at 60°/s decreased. However, neither the multi-ingredient supplement nor whey protein conferred any additional benefit to performance.

The role of dietary supplementation in enhancing physical adaptations to exercise has been extensively examined in both resistance and endurance training [11–13, 15, 25]. However, research on supplementation in the context of HIFT is limited. This may be attributed to the fact that HIFT has only recently become popular among athletes and the general population [1, 2]. To our knowledge, only two other studies have investigated supplementation combined with HIFT [8, 9]. In the first study [8], a multi-ingredient supplement containing ω3 and ω6 fatty acids and antioxidant vitamins was compared with placebo; in the second study [9], egg white was compared with whey protein and maltodextrin. The results of these two studies showed that, although HIFT effectively improved muscle strength and cardiorespiratory fitness, supplementation had no significant impact on these adaptations.

Furthermore, research on the effects of fish protein supplementation on physical performance is limited. Available data indicate that fish protein is a high-quality protein with a favorable essential amino acid profile [16, 17]. Therefore, future studies should compare fish protein with established bioactive protein sources, such as whey protein, to better elucidate the potential effects of fish protein supplementation on muscle health and functional outcomes [17].

The present study evaluated a multi-ingredient supplement containing fish protein, vitamin D, and ω3 fatty acids against the same comparators as in the study cited above [9]. Our results, like those of the previous two studies [8, 9], suggest that multi-ingredient supplements and whey protein do not provide additional benefits to physical performance during HIFT. These findings differ from the conclusions of meta-analyses by O’Bryan et al. [11] and Naclerio and Larumbe-Zabala [30], stating that multi-ingredient supplements had a beneficial effect on strength, albeit in the context of resistance training. Notably, Naclerio and Larumbe-Zabala [30] specifically highlighted creatine as a common additive in these formulations. This difference between our findings and those of the meta-analyses may be due to the lower resistance exercise content of our mixed training program compared with a pure resistance training program. Another possible explanation is that our participants, being young and physically fit, may have limited room for performance improvement with multi-ingredient protein supplementation, as noted by O’Bryan et al. [11]. In addition, factors such as adequate dosage, exercise duration, and sample size may have played important roles in the observed differences.

In the present study, participants received daily 1.6 g of protein/kg body mass, 51 μg of vitamin D, and 1.6 g of EPA and DHA from both the diet and multi-ingredient supplement. These intakes are close to, or consistent with, recommended values reported in the literature [11, 12, 18, 23]. Nevertheless, no effect of supplementation or interaction of supplement and training was observed. This lack of effects may be partly explained by the fact that supplementation strategies specifically in the context of HIFT have not been extensively examined, and evidence-based dosage recommendations for this training modality are currently lacking. However, based on the available evidence, including a study using much higher daily doses of ω3 fatty acids (i.e., 6.9 g ω3 fatty acids in Posnakidis et al. [8]), it seems that the lack of effects is not primarily due to insufficient dosage; instead, other factors may be at play.

Regarding training duration, Wang et al. [1] reported that at least 4 weeks of HIFT with a minimum of 2 sessions per week are required to induce significant improvements in physical fitness and sport-specific performance. However, due to the dearth of literature examining supplementation specifically during HIFT, the optimal intervention duration for observing supplementation effects remains unclear. Therefore, the present study followed recommendations from the resistance-training literature, which suggest that intervention periods of at least six weeks are required to detect potential effects of supplementation [11]. Moreover, Posnakidis et al. [8] did not observe any significant changes with supplementation despite a longer intervention duration (8 weeks). Taken together, these findings indicate that further research is needed to examine whether intervention duration may modulate the potential effects of supplementation on adaptations to HIFT.

In terms of sample size, this was determined a priori and was sufficient to detect medium effects, as defined in the study design. In the present study, 25 participants were initially enrolled and, although four participants withdrew during the intervention, the final sample size remained above the minimum required number. Importantly, the observed correlations between repeated measures across intervention periods were higher than those assumed in the a priori sample size calculation (observed mean *r* ≈ 0.65 vs. assumed* r* = 0.5). These findings further support the robustness of the crossover design and the reliability of the repeated-measure comparisons, without necessitating post-hoc justification of statistical power.

Turning to the effects of exercise training, and consistent with our previous study using the same HIFT intervention [9], the progressive increase in workout duration across the training period likely reflects enhanced exercise tolerance and an improved ability to sustain repeated efforts to exhaustion. Importantly, this occurred without concomitant changes in average HR, suggesting improved cardiovascular efficiency, as also reported previously [9].

Concerning the lack of improvement in VO_2_max in the present study, several studies have reported comparable findings following HIFT [9, 42–44]. However, other research has shown increases in VO_2_max in both trained and inactive individuals after similar interventions [8, 45–47]. These conflicting results may be due to differences in the participants’ training status, duration of the training programs, and training protocols. However, no single factor fully explains the divergence in findings.

The progressive increase in workout duration may be linked to the improvements in upper-body strength and core endurance, as participants gradually performed more repetitions of each exercise, often with greater resistance. In line with previous HIFT studies [9, 44, 48], gains in 1RM and maximal force in the shoulder press were observed. Karpouzi et al. [9] assessed the same exercise, whereas DeBlauw et al. and Held et al. [44, 48] examined 1RM in the barbell overhead press, an exercise involving similar muscle activity. Improvements in core endurance were also consistent with the broader HIFT literature across diverse populations and intervention durations [6, 8, 9, 47, 49, 50].

In contrast, changes in lower-body strength, assessed via isokinetic testing, were minimal. The small decrease (2%) in peak torque of knee extensors at 60°/s may fall within the typical measurement error of isokinetic dynamometry and should therefore be interpreted with caution. Similar findings, including minimal or no changes in peak torque of knee extensors and flexors at 60 and 120°/s following HIFT, have been reported previously [6, 9, 49]. While the multi-joint and functional nature of HIFT may partly explain the limited changes observed in isolated monoarticular assessments, these findings underscore the need for future research employing more functionally relevant assessment methods.

According to the convergence of scientific findings and opinion, a daily protein intake of at least 1.6 g/kg is recommended for maximizing adaptations to resistance training [11, 12, 15]. Additionally, vitamin D and ω3 fatty acid supplementation have shown potential to enhance physical performance (at daily doses of ≥ 20 μg and ~ 2 g, respectively) [20, 21, 24, 27, 28]. This set of knowledge formed the basis of our hypothesis that increasing the daily intake of protein (from 1.0 to 1.6 g/kg), vitamin D (from 30 to 51 μg), and ω3 fatty acids (from 0.2 to 1.6 g EPA and DHA) would enhance the effects of HIFT on the study’s outcome measures. Nevertheless, improvements in exercise capacity and performance were elicited only by HIFT, with no additional benefit from supplementation. These results reject our hypothesis. In line with two other studies [8, 9], a daily protein intake of around 1 g/kg, together with adequate intakes of dietary ω3 fatty acids and vitamin D, appears to be sufficient to support adaptations to HIFT in recreationally trained individuals.

## Limitations

Participants were not blinded to supplementation due to unavoidable differences in taste, smell, and physical form of the supplements. Although outcome assessors, investigators, HIFT instructors, and supplement distributors were blinded, the lack of participant blinding may have introduced performance or expectation bias and should therefore be considered when interpreting the results. Additionally, the nutrient content of the supplements was taken from the manufacturers’ specifications and was not independently verified. Another limitation is that vitamin D washout was not evaluated in the pilot studies, unlike protein and ω3 fatty acid washout; thus, residual effects from vitamin D supplementation cannot be excluded.

Participants were recruited from gym clients, acquaintances, and website postings. The use of convenience sampling may limit the representativeness of the sample and the generalizability of the findings to the wider population of recreationally trained individuals. Moreover, dietary compliance was monitored through weekly self-reported communication with participants, without the use of objective biomarkers (e.g., vitamin D or ω3 fatty acid), which may have introduced reporting bias. Although adherence to the prescribed dietary plans was moderate (75 ± 11%), it did not differ significantly between supplements. Nevertheless, incomplete adherence, together with the absence of objective biomarkers to verify compliance and baseline dietary intake, may have introduced uncontrolled variability in dietary intake, potentially influencing training adaptations, and should be considered when interpreting the results.

## Conclusions

Six weeks of HIFT improved some training and performance variables in healthy, recreationally trained men and women. Thus, HIFT is an effective training method for promoting fitness adaptations. However, increasing the intake of protein, vitamin D, and ω3 fatty acids did not affect the study outcomes. This suggests that a modest daily intake of these nutrients, in the context of a balanced and isoenergetic diet, may be sufficient to support the positive effects of HIFT.

## Data Availability

The data supporting the findings of this study are available in the Hellenic Academic Research Data Management Initiative (HARDMIN) at [https://hardmin.heal-link.gr/dataset/067f042e-4315-4458-b948-a005da9833da].
